# Complexities and protein complexes in the antimycin A-sensitive pathway of cyclic electron flow in plants

**DOI:** 10.3389/fpls.2013.00161

**Published:** 2013-05-27

**Authors:** Dario Leister, Toshiharu Shikanai

**Affiliations:** ^1^Department Biology I, Plant Molecular Biology (Botany), Ludwig-Maximilians-University MunichMunich, Germany; ^2^PhotoLab Trentino - A Joint Initiative of the University of Trento (Centre for Integrative Biology) and the Edmund Mach Foundation (Research and Innovation Centre)San Michele all'Adige and Mattarello, Italy; ^3^Department of Botany, Graduate School of Science, Kyoto UniversityKyoto, Japan

*Antimycin A-sensitive cyclic electron flow (AA-sensitive CEF) was discovered by Arnon and co-workers more than 50 years ago and serves to recycle electrons from ferredoxin (Fd) to plastoquinone (PQ). A role in AA-sensitive CEF has been attributed to the two thylakoid proteins PGR5 and PGRL1 ever since their identification, but this assignment remains controversial. While current technical limitations have prevented unequivocal clarification of their precise function in CEF in vivo, recent biochemical experiments have implied that PGRL1/PGR5 complexes possess Fd-PQ reductase (FQR) activity in vitro. Consequently, PGRL1-PGR5 complexes in flowering plants appear to shuttle between photosystem I (PSI) and the cytochrome (Cyt) b_6_f complex, whereas in the green alga Chlamydomonas PGRL1 (but not PGR5) has been detected in a PSI-Cyt b_6_f supercomplex that has intrinsic CEF activity*.

## A brief look back

**Figure 1:**
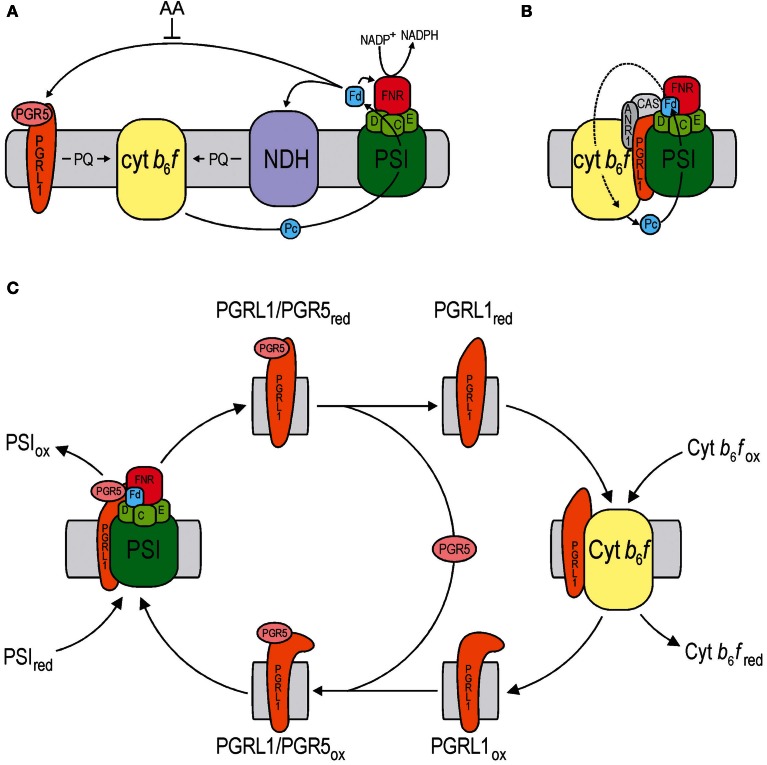
**Model of the role of PGRL1/PGR5 in CEF. (A)** A schematic representation of CEF around PSI in vascular plants, which operates via two partially redundant pathways (Munekage et al., 2004), an NDH-dependent and the PGRL1/PGR5-dependent pathway. Only the latter is inhibited by AA. **(B)** In the green alga Chlamydomonas, CEF can be mediated by a PSI-Cyt *b*_6_*f*-PGRL1-ANR1-CAS supercomplex (Terashima et al., 2012). **(C)** Model for the FQR activity of PGRL1/PGR5 according to Hertle et al. (2013). Fd, ferredoxin; Pc, plastocyanin; PQ, plastoquinone.

Cyclic electron flow (CEF) around photosystem I (PSI) recycles electrons from Fd to PQ, generating ATP without accumulation of NADPH (reviewed in: Shikanai, 2007) (see Figure 1A). The discovery of CEF dates back to 1954, when Arnon and co-workers identified, in isolated chloroplasts, a process that generates ATP without production of O_2_ (Arnon et al., 1954), requires Fd and is sensitive to antimycin A (AA) (Tagawa et al., 1963). The Arnon group then extended their work to the identification of another type of electron flow, namely non-cyclic (or linear) electron flow (LEF) (Arnon et al., 1958), which is now widely accepted as the major pathway for the light reactions of photosynthesis. Today, investigation of the function of the LEF machinery has reached the atomic level (Umena et al., 2011), but the molecular details and physiological significance of CEF remain largely elusive—a situation which is largely due to technical constraints on our ability to measure CEF directly, especially in leaves (reviewed in: Johnson, 2005). Indeed, while the scepticism encountering the concept of ATP production in chloroplasts has disappeared over the years (Arnon, 1991), doubts about the validity of the concept of CEF persist.

## The genetic evidence

A breakthrough in CEF research was achieved with the discovery that a multiprotein complex, which resembles Complex I in the mitochondrial respiratory chain, mediates CEF in cyanobacteria (reviewed in: Ogawa and Mi, 2007). This made the equivalent complex in chloroplasts, the so-called “NAD(P)H dehydrogenase” or NDH complex, a prime candidate for a component of CEF in algae and plants. Analysis of knockout mutants of plastid *ndh* genes in tobacco then showed that chloroplast NDH indeed mediates electron transport from stromal reductants to PQ, although photosynthesis is not overtly affected in these mutants, at least under greenhouse conditions (Burrows et al., 1998; Shikanai et al., 1998). Chloroplast NDH most probably accepts electrons from Fd rather than NAD(P)H (Yamamoto et al., 2011), but it is insensitive to AA (Endo et al., 1997). Consequently, Arnon's CEF cannot be mediated by the NDH complex and, to avoid confusion, we will refer to the process originally described by Arnon as “AA-sensitive CEF.”

AA-sensitive CEF was finally “re-discovered” in the course of a screen for Arabidopsis mutants defective in the induction of non-photochemical quenching (NPQ) at high light intensity (Munekage et al., 2002). In the mutant *proton gradient regulation 5* (*pgr5*), the PSI reaction Centre (P_700_) is highly reduced in the light, whereas it is oxidized in the wild type (Munekage et al., 2002). This P_700_ phenotype is probably due to decreased production of ATP, which leads to photoinhibition of PSI. Soon after that report, the Arabidopsis *chlororespiratory reduction* (*crr*) mutants, which are defective in NDH activity, were isolated in a screen based on chlorophyll fluorescence imaging (Hashimoto et al., 2003). Although both *pgr5* and *crr* single mutants behave like wild type under standard conditions, growth and photosynthesis are severely impaired in *crr pgr5* double mutants (Munekage et al., 2004).

To demonstrate that the *pgr5* and *crr* mutants are indeed defective in CEF, an assay was employed in which Fd-dependent PQ reduction activity was measured in ruptured chloroplasts. With this assay, clear and additive effects on chlorophyll fluorescence were observed for the *crr* and *pgr5* mutants, and were ascribed to lesions in the NDH- and AA-sensitive pathways of CEF, respectively (Munekage et al., 2002, 2004). However, these conclusions are based on phenotypical analyses of the relative severity of the CEF lesion in the mutants concerned, and are thus constrained by the methodological limitations mentioned above. Hence, in the absence of clear phenotypic criteria for deciding when a photosynthetic mutant qualifies as a true AA-CEF mutant, an alternative and more indirect role for PGR5 [and its interacting partner PGRL1 (DalCorso et al., 2008)] in CEF has been proposed (see: Nandha et al., 2007).

However, it is worth pointing out here that, irrespective of the status of the CEF measurements, the following major conclusions derived from the analyses of the *pgr5* mutant remain valid: (1) PGR5 and PGRL1 are necessary for NPQ induction and protection of PSI from photoinhibition (Munekage et al., 2002; DalCorso et al., 2008). (2) The *pgr5* mutant is sensitive to fluctuating light levels (Suorsa et al., 2012) and the *crr pgr5* double mutant is sensitive even to constant low light (Munekage et al., 2004). (3) In the ruptured chloroplast assay, AA affects chlorophyll fluorescence in the WT but not in *pgr5* plants (Munekage et al., 2002, 2004). Taken together, both the role of PGR5 in photoprotection (1 + 2) and its relationship to sensitivity to AA (3) provide a reasonable basis for the hypothesis that PGR5 is involved in AA-sensitive CEF.

## Biochemical analyses

The transmembrane protein PGRL1 was identified in thylakoids of Arabidopsis by DalCorso et al. (2008). Because plants lacking PGRL1 showed a perturbation of CEF similar to that seen in PGR5-deficient plants, the protein was named “PGR5-like protein 1” or PGRL1. Yeast two-hybrid and split-ubiquitin assays, as well as PSI co-purification experiments demonstrated that PGRL1 and PGR5 interact with each other and with PSI. PGRL1 also interacts with Fd, the Fd-NADPH oxidoreductase (FNR) and the cytochrome (Cyt) *b*_6_*f* subunit Cyt *b*_6_—at least in yeast assays. Moreover, mutants lacking PGRL1 do not accumulate PGR5, whereas PGR5-less plants still express PGRL1, suggesting that PGRL1 represents the docking site for PGR5. Based on these data, it was proposed that the PGRL1-PGR5 complex, together with Fd and the FNR, represents the long-sought Fd:PQ reductase (FQR) and facilitates AA-sensitive CEF in plants, while spectroscopic data were also adduced in support of a regulatory role for the complex (DalCorso et al., 2008). In the green alga Chlamydomonas, PGRL1 functions in iron sensing (Petroutsos et al., 2009) in addition to regulating CEF in conjunction with ANR1 and CAS, and all three are associated with each other in a multiprotein complex (Terashima et al., 2012; see Figure 1B).

In a more recent study, PGRL1 was found to be a redox-active protein that exists in monomeric and homodimeric forms, and as a heterodimer with PGR5 (Hertle et al., 2013) (see Figure 1C). Moreover, co-immunoprecipitation assays confirmed the interaction of PGRL1 with Cyt *b*_6_. Systematic mutagenesis of six conserved cysteine residues in PGRL1 identified three functional redox-regulated domains: a homodimerization motif that is regulated by thioredoxins, an iron-containing cofactor site and a domain required for heterodimerization with PGR5. *In-vitro* assays showed that the PGRL1–PGR5 complex can accept electrons from Fd, whereas PGRL1 alone is sufficient to reduce quinones. In addition, the redox kinetics of PGRL1 *in planta* confirm that it receives electrons from PSI in a PGR5-dependent manner. Taken together, these results strongly argue that PGRL1 acts as the plant FQR, with PGR5 playing an accessory but essential role (Hertle et al., 2013).

## Interspecific diversity

The function of the PGRL1/PGR5-dependent CEF pathway seems to be conserved in flowering plants, as indicated by the phenotype of PGR5 knock-down lines of rice, which in many respects resembles that of Arabidopsis *pgr5* mutants (Nishikawa et al., 2012). In contrast, cyanobacteria appear to lack the PGRL1 protein. However, inactivation of a cyanobacterial gene that displays weak homology to PGR5 appears to perturb cyanobacterial AA-sensitive CEF (Yeremenko et al., 2005). Homologues of both PGR5 and PGRL1 exist in the green alga *Chlamydomonas reinhardtii*. However, in contrast to the situation in land plants (where a PSI-Cyt *b*_6_*f* supercomplex has not been detected yet), in Chlamydomonas PGRL1 is part of a supercomplex that contains PSI and the Cyt *b*_6_*f* complex (Iwai et al., 2010) (see Figure 1B), together with the algal-specific protein ANR1 and the protein CAS, which is conserved in algae and vascular plants (Terashima et al., 2012). Moreover, (1) Chlamydomonas CEF appears not to be sensitive to AA (Iwai et al., 2010), and (2) as in other species of Chlorophyceae, *ndh* genes are absent in Chlamydomonas. Interestingly, the Chlamydomonas PGR5 protein has yet to be detected in the PSI-Cyt *b*_6_*f*-PGRL1-ANR1-CAS supercomplex.

One may therefore speculate that PGRL1/PGR5-dependent CEF evolved from a process that requires PGR5 only (Synechocystis) to one that entails cooperation between the two proteins—in the context of either an AA-insensitive supercomplex (Chlamydomonas) or an AA-sensitive heterodimer (Arabidopsis).

## Controversies

The role of PGR5/PGRL1 in CEF is still very much a live issue. We will now review the arguments advanced against the involvement of PGR5/PGRL1 in AA-sensitive CEF.

1. Doubts have been raised regarding the reliability of the ruptured chloroplast assay (see above) as an indicator of CEF rates *in vivo* (Nandha et al., 2007). The kinetics of chlorophyll fluorescence responses observed in this assay are certainly too slow to reflect *in-vivo* rates of CEF directly. But this feature, which can plausibly be attributed to functional impairment of ruptured relative to intact chloroplasts, need not necessarily exclude the possibility that the assay provides an indirect measure of CEF. In fact, the additivity of the effects of *crr* and *pgr5* mutations, together with the impact of AA, on chlorophyll fluorescence kinetics indicate that this assay, at least qualitatively, captures a process that is closely linked to CEF.
2. Results from P_700_ oxidation kinetics and certain chlorophyll fluorescence measurements done on leaves have been taken as evidence against a direct involvement of PGR5 in CEF (Nandha et al., 2007). However, these techniques also do not measure CEF directly, and some of them fail to detect an inhibitory effect of AA on CEF (Joliot and Joliot, 2002).
3. Avenson et al. (2005) monitored electrochromic shift (ECS) signals and found that the absence of PGR5 affects a flux of protons corresponding to ~13% of that from LEF. This finding can be explained in two ways. Either AA-sensitive CEF contributes around 13% to the total proton motive force (*pmf*) (Avenson et al., 2005), or the total contribution of CEF is markedly higher than 13% and CEF is only partially suppressed in *pgr5* mutants, which would point to a regulatory role of PGR5/PGRL1 in CEF (Nandha et al., 2007). Here the same objection can be raised as before: like the ruptured chloroplast or P_700_ kinetics assays discussed above, the ECS approach itself does not directly monitor the rate of CEF but the *pmf*, which is the primary product of CEF. Puzzlingly, proton conductivity (g_H^+^_) is increased in Arabidopsis *pgr5* (Avenson et al., 2005) and rice *PGR5* knockdown lines (Nishikawa et al., 2012). It seems unlikely that PGR5 directly regulates the ATPase, but perhaps ATP production is somehow upregulated to compensate for the lack of CEF in *pgr5* plants (Nishikawa et al., 2012).
4. Suorsa et al. (2012) have suggested that PGR5 protects PSI from photoinhibition by regulating LEF, in particular Cyt *b*_6_*f* activity. This idea actually does not conflict with the concept that PGRL1/PGR5 mediates CEF. A tentative 13% contribution of PGRL1/PGR5-dependent CEF to total *pmf* is not insignificant and the direct CEF-related effect of the *pgr5* mutation on lumen pH might be exacerbated by the altered regulation of proton conductivity (see above). In consequence, PGR5 could regulate Cyt *b*_6_*f* indirectly via its primary effect on lumen acidification during CEF.

## Conclusions

We currently lack a reliable way of measuring CEF directly, and thus have no means of clearly distinguishing between partial and complete lack of CEF. Conversely, without mutants that are generally acknowledged to lack CEF specifically, it is impossible to establish and calibrate methods that are suitable for quantifying CEF. Therefore, a crucial step in elucidating AA-sensitive CEF will be to corroborate the function of PGRL1 and PGR5 as an FQR and address the basis for the apparently different functions of PGRL1 and PGR5 in plants and green algae.
